# Do open educational resources improve student learning? Implications of the access hypothesis

**DOI:** 10.1371/journal.pone.0212508

**Published:** 2019-03-06

**Authors:** Phillip J. Grimaldi, Debshila Basu Mallick, Andrew E. Waters, Richard G. Baraniuk

**Affiliations:** OpenStax, Rice University, Houston, Texas, United States of America; National University Ireland Galway, IRELAND

## Abstract

Open Educational Resources (OER) have been lauded for their ability to reduce student costs and improve equity in higher education. Research examining whether OER provides learning benefits have produced mixed results, with most studies showing null effects. We argue that the common methods used to examine OER efficacy are unlikely to detect positive effects based on predictions of the access hypothesis. The access hypothesis states that OER benefits learning by providing access to critical course materials, and therefore predicts that OER should only benefit students who would not otherwise have access to the materials. Through the use of simulation analysis, we demonstrate that even if there is a learning benefit of OER, standard research methods are unlikely to detect it.

## Introduction

The textbook has long been a critical component of the education system at all levels. In addition to providing a scaffold for content discussed in a course, textbooks have historically been the primary learning resource for students. For a variety of market-based reasons, the price of textbooks has risen dramatically over the last two decades, outpacing the price increases of all goods and services by almost four times [1]. Within higher education, these price increases ultimately fall on the students, who are responsible for procuring their own course materials. In response to these price trends, many educators have turned to open educational resources (OER) [2, 3]. While OER refers to any educational resource that is openly licensed and freely distributed, for the purposes of this document we will limit our discussion to OER textbooks. Over the last decade, OER has risen dramatically in popularity. According to OpenStax, the leading producer of OER textbooks, adoption of OER textbooks has saved students an estimated $500 million dollars since 2012 [4]. Moreover, recent survey data [5] suggest that OER textbooks now rival commercial textbooks in terms of overall market share. More importantly, textbook prices appear to have recently leveled off for the first time in three decades, an effect which is partially attributed to increased competition from OER alternatives [6].

While the OER movement has been successful in reducing the cost of educational materials, many have wondered whether adoption of OER affords additional benefits, such as improved student learning outcomes [7]. This question has motivated a flurry of empirical research comparing the grades of students who used OER textbooks to students who used a commercial textbook (for a recent review, see [8]). Overall, this research has produced somewhat mixed results. Several studies have found no significant differences between OER and traditional textbooks on student grades [9–12]. Occasionally, however, negative or positive effects are found. One study [13] found no significant difference in regular exam scores, but did find a benefit of OER adoption on a specialized exam score. Another study [14] compared OER and traditional texts across seven high school classes and found a negative effect of OER in two classes, and no significant difference in the other five classes. In a study comparing OER and a commercial textbook across fifteen courses [15] a negative effect of OER was found in one course, a positive effect of OER in five courses, and a non-significant difference in the remaining nine courses. A six-semester study comparing OER to non-OER [16] observed a negative effect in two semesters, and a positive effect in one semester. However, a later analysis revealed these effects were likely artifacts of confounding variables. A study comparing digital and print OER books to traditional print text across three course exams [17] found a positive effect of digital OER on only one exam. A large scale evaluation of OER [18] found positive effects of OER adoption on student grades. It is worth noting that this research varies considerably in terms of quality and rigor. Nearly all used quasi-experimental designs, and some failed to control for possible confounding variables (e.g., [18]; see [19] for a discussion). Nevertheless, the important thing to note is that the majority of comparisons in the literature find null effects of OER adoption on learning outcomes.

Why do most comparisons of OER to traditional materials fail to find a positive effect of OER? On one hand, the primary goal of OER is to offer an alternative to commercial textbooks that are comparable in quality, but free and openly licensed. Assuming an OER textbook is no different in quality, then there are no meaningful differences to explain effects on learning outcomes. License and cost certainly should not affect learning at a cognitive level. In this sense, the frequency of null effects is expected. On the other hand, the price of a textbook can affect whether a student decides to purchase a textbook, and a student cannot learn from a textbook they do not have. If we reasonably assume that having a textbook is better for student learning than not having a textbook, these students would then be at a learning disadvantage. Thus, adoption of OER would be effective as a learning intervention because it ensures that all students have access to the textbook, and would therefore result in better learning outcomes (for similar discussion, see [8, 15, 18]). We refer to this idea as the *access hypothesis*.

If access is the primary mechanism for how OER might affect learning outcomes, then we can see that current research approaches are not well suited for detecting an effect of OER adoption. In most educational research, an intervention is expected to impact all students who receive the intervention, and its impact is measured by comparing students who receive the intervention to students in a control condition. However, the access hypothesis predicts that an OER intervention should only affect a subset of students—specifically those who would not otherwise have access to the textbook. Students who are willing or able to purchase the textbook should not be affected. Yet, every study that has evaluated OER efficacy to date has treated OER as any other intervention, specifically by comparing an entire sample of students who received the OER intervention to a sample of students who did not. Indeed, this is the approach recommended by the most active researchers of the field [20]. The problem with this approach is that the effect of the intervention is washed out by students who are not expected to be affected by the intervention. To draw an analogy, the current research approach in OER is the equivalent of measuring the effect of a pain relieving drug on a sample of people who are mostly not in pain. In this sense, we should not expect to observe effects of an OER intervention, even if we believe that having access to a textbook is beneficial to learning.

If the impact of OER is measured across an entire sample of students, then it is necessary for researchers to consider the textbook access rates prior to implementation of OER. Past research reveals some insights as to what the expected textbook access rates are in a typical classroom. A recent survey of over 22,000 Florida students enrolled in public universities and colleges found that close to 66.5% of students reported not purchasing a textbook at some point in their academic career [21]. While this statistic is concerning, the data are limited in that they do not indicate what the access rates are in any given classroom. Just because a student avoids purchasing a textbook once does not mean they will repeat the behavior for all of their classes. Indeed, more targeted research reveals that access rates can be very high. A survey of 824 University of Colorado at Boulder Physics students [22] found that 97% purchased the required texts. Another survey of 1023 students at an undisclosed university across a range of introductory level science courses [23] found that 96% of students reported purchasing their required texts. A survey of 162 students in a political science course [12] found a 98% access rate. We can imagine that if an OER intervention were conducted on these samples, it would be very difficult to observe a positive effect because the existing access rates are already so high. Of course, we cannot expect access rates to be high in every classroom. An internal survey at Virginia State University [24] reported that only 47% of students purchased textbooks. Unfortunately, they did not report how many students were included in this sample. Regardless, it is fair to say that the rate of textbook access will vary across contexts and student populations. As we will see, the access rate of any given population can have a profound effect on the results of research aimed at evaluating the impact of OER adoption.

In this paper, we argue that the standard approach taken in past research on OER efficacy is severely limited in its functional ability to properly evaluate the impact of OER. This functional limitation is controlled by the existing textbook access rate prior to an OER intervention. In order to formally illustrate this point, we conducted a series of simulated experiments designed to mimic a typical study on OER effectiveness. We used these simulated experiments to measure the likelihood of a standard OER efficacy study to correctly reject the null hypothesis (i.e., statistical power [25]). A simulation study is useful because we can examine the expected results of an experiment in perfectly controlled conditions. Most real world educational research is plagued by instructor artifacts, confounding variables, and random differences between groups. Moreover, it is incredibly difficult to implement a randomized control trial with real students. In a simulation, we do not have to worry about any of these constraints. A simulation is also necessary in this case, because traditional power analysis does not allow us to vary the number of students who might be affected by an intervention, as is predicted by the access hypothesis. The primary goal of these simulations was to examine the influence of access rate on statistical power in a typical study of OER effectiveness, and make inferences about the likelihood of detecting a positive effect of OER adoption in a real world study. We then apply this model to evaluate existing studies that have already been conducted.

## Simulation Study

### Methods

#### Design

**Fig 1:**
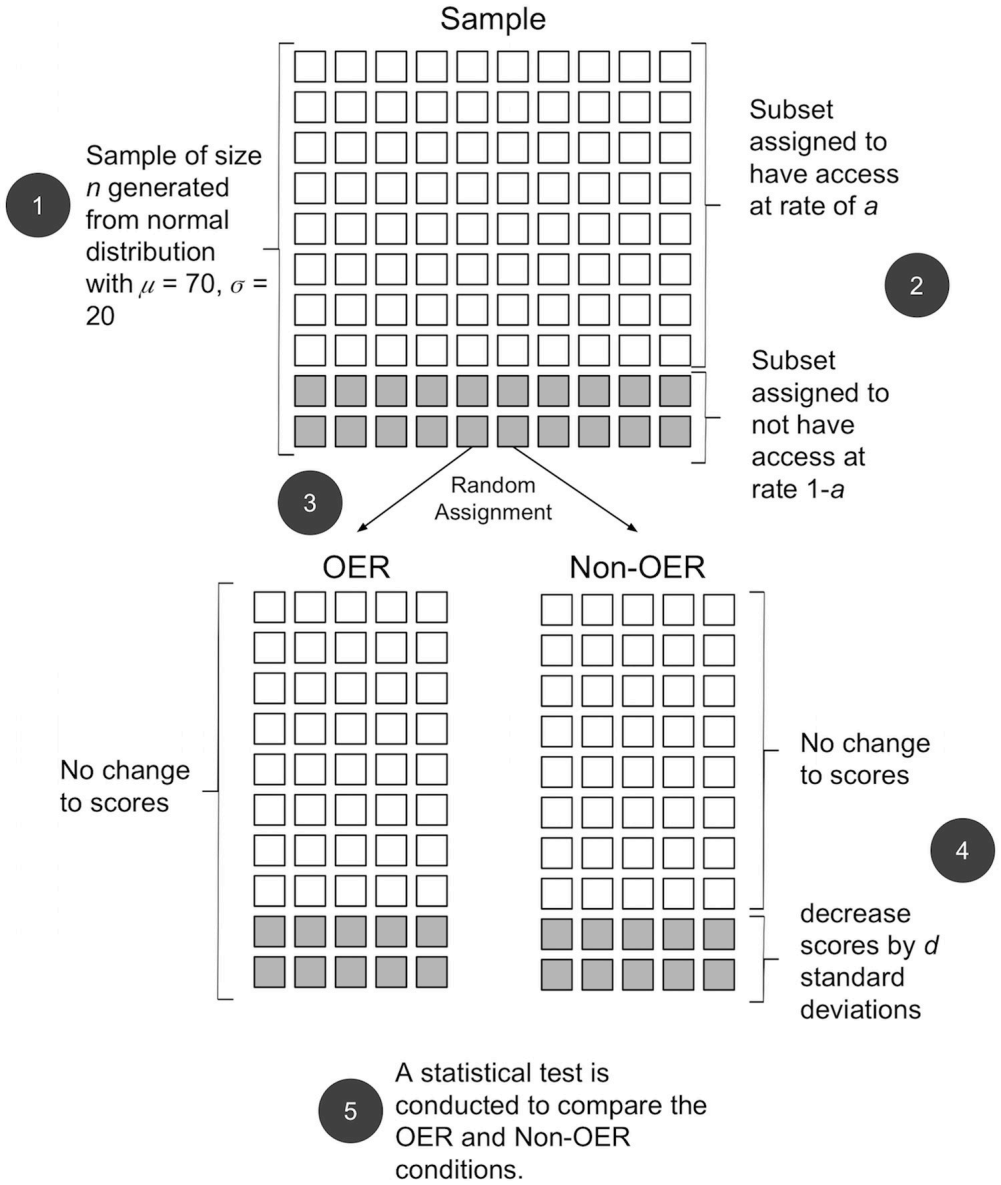
Overview of the simulated experiments. For each experiment, a sample of student scores were generated, and students were determined to have access or not to the textbook. Students were then randomly assigned to either an OER or Non-OER condition. The effect of access was simulated by reducing scores for students determined to not have access, but only in the Non-OER condition. Lastly, a statistical test the OER and Non-OER conditions was performed. See text for more information.

For each simulated experiment, we first generated a sample of *n* student scores *s* from a normal distribution *s* ∼ *N*(*μ*, *σ*^2^), truncated between 0 and 100. These scores represented the final grade of each student in the course on a 100 point scale, where *μ* was the sample mean and *σ* was the sample standard deviation. Second, students were randomly determined to have access to the textbook at a rate of *a*, and not have access at a rate of 1 − *a*. Third, students were randomly assigned to either an OER or Non-OER condition with the constraint that both conditions must have an equal size. Fourth, in order to simulate the effect of access, we decreased the score of the students in the Non-OER condition who were previously determined to not have access to the textbook. The scores of students in the OER condition were unaffected, representing the fact that all of these students now have access to the book. The magnitude of the score decrease was equivalent to *dσ*. The parameter *d* represents the effect size [26] of having access to a textbook. Finally, we fit a regression model that predicted student score by condition, and tested the condition coefficient against 0 by using a standard t-test. An overview of the simulation is shown in Fig 1.

#### Parameter values

For determining the value of *n*, we wanted to use similar sample sizes as studies that have examined OER in past research. Of the 42 direct comparisons of OER and non-OER materials on course grade [9, 12, 14–18, 27, 28], 95% involved sample sizes smaller than 5000 students. Thus, we examined levels of *n* between 100 and 5,000. We address sample sizes larger than 5,000 later in this report.

For generating our sample distribution, we set *μ* to 70 and *σ* to 20. We chose these values because we felt they were representative of a typical classroom, and similar to those we have observed in past research. However, because the effect of an intervention is measured by relative differences in scores, the actual values used here do not have much influence on the outcome of the simulation.

For the *a* parameter, which represents the proportion of students who are expected to have access to the textbook, we wanted to examine access rates that would be expected in a typical college classroom. We examined 6 different levels of *a* − 40%, 50%, 60%, 70%, 80%, and 90%. This range is likely to cover most student populations that might appear in OER research.

The *d* parameter represents the effect of having access to a textbook versus not having access to a textbook. We anticipated the literature would provide a clear direction for setting this parameter. To our surprise, despite the ubiquity of textbooks in higher education, there are few studies examining the effects of textbooks in general (both OER and non-OER) on learning outcomes. To our knowledge, there are no experimental studies that would afford calculation of a reasonable effect size of textbook usage. There are some correlational studies that at least show positive relationships between textbooks and learning. One study [22] reported moderate correlations between the amount of reading assignments a student completed and their final course grade for conceptual physics courses (*r* = .45), but no correlation for calculus based physics (*r* = .07). Another study [23] found that students who reported regularly reading their textbook had higher grades than students who read their textbooks only occasionally. However, they also found no difference between students who *never* read their books and those who read regularly. A positive relationship between student grades and engagement was found between student grades and engagement with a digital textbook, even after accounting for general student aptitude [29]. In sum, these studies show at the very least that use of the textbook can be beneficial to learning. We concluded that if there is an effect of textbook access on learning, it is likely to be small. Thus, we set *d* to a value of 0.25, which is considered to be the minimum effect size necessary for an educational intervention to be substantively important [30].

#### Procedure

For each level of *n* and *a*, we repeated the experiment 10,000 times and recorded the *p*-values of each experiment. Statistical power was computed as the proportion of studies with *p*-values lower than *α*. All simulations were conducted using R [31], and based on code presented in [32]. The full code used for the simulations is available on GitHub (https://github.com/openstax/oer-simulation-study).

### Results

**Fig 2:**
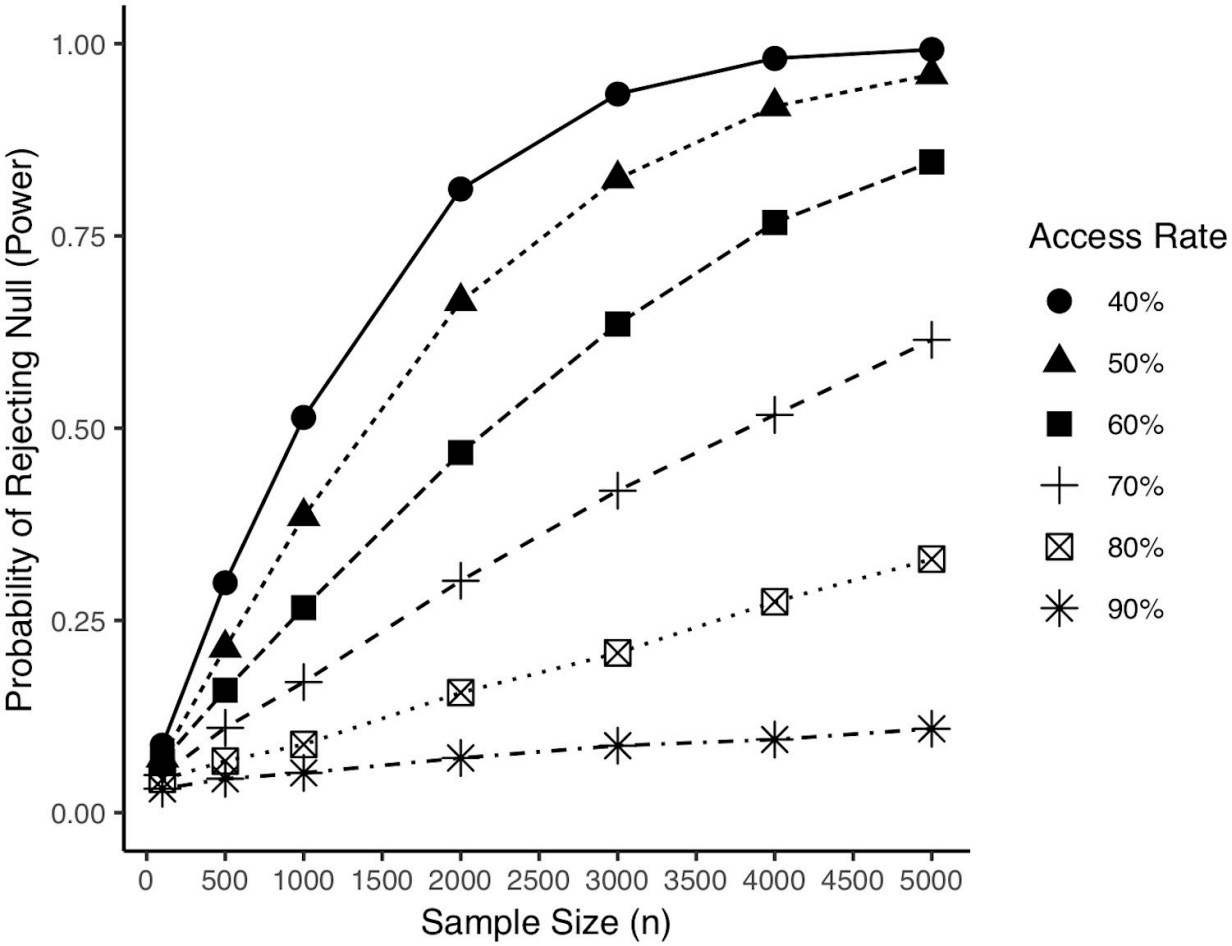
Probability of successfully detecting an effect of OER as a function of access rate and sample size. A study sample’s access rate to textbooks prior to adopting OER can severely hinder the likelihood of detecting an effect of OER, even at large sample sizes.

We examined the proportion of simulated experiments that rejected the null hypothesis at the standard *α* of.05 (i.e., power). The results are shown on Fig 2. As is the case of all experiments where samples are drawn from normal distributions, the probability of success increases with *n* [25]. However, we also see that access rate (*a*) plays a strong influence on the ability to detect the effect of OER. When access is very low, experiments have a much higher likelihood of correctly rejecting the null hypothesis with smaller *n*. This makes sense, because there are more student in the sample that can be impacted by the intervention. However, as *a* increases, it pulls the probability of success down. Indeed, when *a* is large, it requires very large numbers of students to detect a significant effect. To illustrate the strength of this relationship, an OER experiment with 10,000 students will have a 89.3% chance of success when the access rate is 70%. However, the same 10,000 student experiment conducted on a sample with an access of 80% will only have a 56.5% success rate. The situation gets considerably worse when the access rate is 90%. This experiment would only have a 19% success rate. The fact that an experiment with 10,000 students would have such a low chance of correctly rejecting the null hypothesis demonstrates the influential role of access rate.

## Examination of past studies

The results of these simulations beg the question—how are we to interpret previous studies that have examined the effects of OER interventions on learning outcomes? To this end, we used the simulation procedure described previously to conceptually replicate prior studies on OER efficacy, with the goal of estimating the probability that such a study would have detected an effect of OER, given the reported sample sizes used in those studies at different levels of access.

### Methods

#### Selection of research

From the literature, we were able to find 42 direct comparisons of OER to traditional materials, across 9 publications [9, 12, 14–18, 27, 28]. We did not include a study or comparison if tests of statistical significance were not reported. Further, we only included comparisons that used a continuous performance metric as their dependent variable (i.e., grades on a 0-4 scale or test scores). Comparisons that used non-performance based dependent variables (e.g., drop or withdrawal) were not included, as they are not suitable for use as measures of learning. Some studies (e.g., [15]) examined both grades *and* pass rates separately, which is a dichotomous version of grade (i.e., C- or better.). As an aside, it is not clear to us why both measures are sometimes used, as the measures are likely highly correlated. In cases where both measures were used, we only included comparisons on course grade. We did not examine studies that only examined pass rates, because these studies use non-parametric statistics which are not applicable to the power analysis we conducted. Also, several studies conducted both an analysis which collapsed across different courses or semesters, and then conducted separate analyses for each of these levels [16, 27]. In these cases, we only included each separate analysis, but not the overall analyses. In the case of [18], they collapsed across multiple courses without conducting separate analysis for those courses. In this case, we only included the overall analysis. The complete list of comparisons is shown on Table 1.

**Table 1:** Estimated power of prior efficacy studies across different access rates (*a*).

	n			Power		
Comparison [Reference]	Non-OER	OER	Outcome	*a*_40%_	*a*_60%_	*a*_80%_
Earth Science [27]	351	359		0.54	0.28	0.11
Biology [27]	486	499		0.70	0.36	0.13
Chemistry [27]	437	416	+	0.64	0.31	0.12
Chemistry [9]	448	478		0.67	0.35	0.13
Biology [15]	134	99		0.22	0.14	0.06
Business [15]	228	227	-	0.39	0.20	0.07
English [15]	93	46	+	0.14	0.10	0.04
Math 60 [15]	722	49		0.20	0.11	0.05
Math 80 [15]	143	20		0.10	0.07	0.04
Math 100 [15]	358	47		0.18	0.10	0.04
Math 150 [15]	76	30		0.11	0.07	0.04
Math 219 [15]	335	27		0.12	0.08	0.04
Math 1010 [15]	4,531	84		0.30	0.15	0.08
Math 1210 [15]	247	93	+	0.26	0.14	0.06
Math 920 [15]	345	42	+	0.16	0.10	0.06
Psych 100 [15]	822	26	+	0.13	0.07	0.04
Psych 101 [15]	814	109		0.33	0.18	0.07
Psych 103a [15]	52	97		0.15	0.10	0.05
Psych 103b [15]	364	91		0.30	0.13	0.06
Writing [14]	4,707	552		0.95	0.64	0.22
Reading [14]	1,553	477		0.88	0.53	0.17
Psychology [14]	1,849	223	-	0.61	0.36	0.10
Business [14]	168	1,070	-	0.50	0.27	0.09
Geography [14]	731	388		0.70	0.40	0.13
Biology [14]	844	323		0.70	0.35	0.13
Algebra [14]	967	221		0.54	0.29	0.10
Summer 2014 [16]	1,416	131	-	0.41	0.22	0.09
Fall 2014 [16]	3,576	226	-	0.62	0.34	0.11
Spring 2015 [16]	2,620	1,250		1.00	0.86	0.32
Summer 2015 [16]	855	675		0.86	0.54	0.18
Fall 2015 [16]	1,951	2,394	+	1.00	0.92	0.46
Spring 2016 [16]	1,070	2,912		0.99	0.83	0.32
Overall [18]	11,681	10,141	+	1.00	1.00	0.97
Exam 1 Open Print [17]	83	51		0.12	0.08	0.04
Exam 1 Open Digital [17]	83	44		0.13	0.08	0.05
Exam 2 Open Print [17]	83	51		0.13	0.07	0.05
Exam 2 Open Digital [17]	83	44		0.11	0.10	0.04
Exam 3 Open Print [17]	83	51		0.12	0.08	0.06
Exam 3 Open Digital [17]	83	44	+	0.13	0.05	0.04
Psychology [28]	316	204	+	0.44	0.23	0.10
Lester’s Class [12]	112	122		0.24	0.12	0.05
Lawrence’s Class [12]	88	56		0.16	0.10	0.04

Note that + indicates the comparison favored the OER condition while - favored the non-OER condition.

#### Simulation power analysis

For each of the prior comparisons, we conducted 10,000 simulations using the same sample sizes reported by the authors. Since there is no way of determining the true access rate of the samples used in these comparisons, we used a range of *a* values (40%, 60%, and 80%). All other assumptions of the prior simulations were the same (*μ* = 70, *σ* = 20, *d* = .25, *α* = .05), with one exception. We noted that many of the prior studies under consideration had imbalanced numbers of Non-OER and OER students, typically with far more Non-OER students than OER students. Rather than assuming equal sample sizes like in the previous simulations, we matched the sample size allocation ratio of the comparison study in the simulations. For example, the study in [15] reported one comparison with 4615 students, but 4531 in the Non-OER condition, and 84 students in the OER condition. In our simulations of this study, we drew samples of 4615 students, and allocated 98.2% to the Non-OER condition, and 1.8% to OER condition.

### Results

The estimated power for each comparison, for access rates of 40%, 60%, and 80%, are shown on the far right columns of Table 1. We can see that for most comparisons, even under the most optimistic of scenarios (i.e., 40% access), the expected likelihood that the comparison would yield a positive significant effect of OER is very small. Only the comparisons which had very large sample sizes had substantial power at the 40% access level [16, 18], though even some of these comparisons had low power at 80% access rates. Note that for many studies, power is so low at the 80% access level that the probability of correctly rejecting the null hypothesis is just as likely as falsely rejecting a true null hypothesis (*α* = .05)! Thus, if there was an 80% access rate, these experiments were just as likely to detect a real effect of OER as they were to detect a false effect of OER. Interestingly, one comparison [15] had low power even with a sample size over 4000 students. This was due to the extreme imbalance of students in the Non-OER and OER conditions.

Given the results of this power analysis, we can determine the expected number of comparisons that should have correctly rejected the null hypothesis by summing the power values for each level of access. Across the 42 reported comparisons, we would only expect to observe significant effects of OER 18, 11.5, or 5.2 times, for the 40%, 60%, and 80% access rates, respectively. Note that only 9 of the 42 comparisons on Table 1 found positive effects of OER on learning outcomes. Even though this number seems very low, the results of the simulation power analysis demonstrate that this is well aligned with what should be expected, even if there is a real effect of OER. Of course, our power estimates assume perfect conditions. These real world studies have many confounding factors to contend with, so it is likely that the real power of these studies was even lower than what we estimated. In this case, it is possible that the number of significant effects found so far could even be higher than what would be expected.

## Discussion

Over the last decade, there has been a fair amount of research examining whether the adoption of OER textbooks improves student learning outcomes relative to commercial textbooks. The majority of this research has found no significant difference between OER and commercial texts when measuring learning performance. We have argued that one possible reason why most tests of OER efficacy fail stems from the predictions of the access hypothesis. The access hypothesis, formally introduced by us, states that OER might improve learning outcomes relative to traditional course materials by improving access to the textbook. Therefore, an OER intervention should only affect a subset of students who would not have access to the textbook. Through the use of simulated power experiments, we have demonstrated that the textbook access rate of a research sample prior to the intervention has profound effects on statistical power. As the access rate of a sample increases, the power of an experiment decreases dramatically. If the access rate is high, even studies with very large sample sizes should produce null results most of the time.

Overall, our analysis helps to provide better context to the studies that have examined OER efficacy. Even under ideal conditions, detecting positive effects of OER should be extremely difficult. The fact that most studies have found null effects is not surprising; in fact, these null effects are expected. Furthermore, our results stress the importance of being skeptical of studies that report positive effects of OER interventions. This is especially true if the study used relatively small sample sizes. In our simulation experiments, even comparisons with 1000 students are more likely to discover null effects than positive ones, even with access rates at the low end of the scale.

### Implications for OER research

These results have several implications for future research in OER. First, we recommend that researchers attempt to measure textbook access rates in their student population prior to implementation of OER. If access rates are very high, it is important to consider that the likelihood of detecting an effect on learning outcomes should be very low. The effect of access rates should be considered when interpreting null results.

Second, it is critical that future research works towards determining the true effect size of textbook access on learning. Determining the true effect size will afford far more reliable power calculations, and more importantly, enable more meaningful interpretation of research studies. For instance, a high powered study that produces a null result is more meaningful than a low powered study that produces a null result, because the null result is unexpected in the case of a high powered study. Unfortunately, accurate measures of power require a reliable measure of effect size, and the vast majority of studies on OER efficacy do not report enough statistics in their analyses for computation of effect size estimates. It is critical that researchers report all relevant test statistics, p-values, sample sizes, means, and measures of dispersion. We encourage reviewers and editors of future research insist that authors report these measures.

Finally, it is common for OER researchers to conduct comparisons without making an explicit hypothesis or prediction. Hypotheses and predictions are critical, because they help guide research designs and interpretation of results. In the case of the access hypothesis, having an explicit mechanism makes it clear that the intervention should only affect some students. We cannot help but wonder if so many low power null effects would have been published had the access hypothesis been formally proposed earlier.

### Potential theoretical mechanisms for OER efficacy

In this paper, we have discussed access as being the primary mechanism for why OER might improve learning. It is certainly possible that adoption of OER could affect learning outcomes in other ways. One idea is that the open nature of OER affords the ability to teach in ways that are not possible given the constraints of closed source materials [7]. Another idea is that OER may provide better or worse quality than the commercial counterpart (e.g., [17]). However, as mentioned previously, these ideas are rarely expressed as a formal hypothesis, and the mechanisms are rarely tested as part of the research. One exception is the work of [17], which compared learning outcomes from an OER and commercial textbook, but also examined the perceived quality and readability of the books. While differences in perceived quality and readability were observed, these differences did not translate into strong benefits to learning [17]. It should be noted that other mechanisms would not be subject to the same power constraints as access, as these mechanisms would presumably affect all students in the study. Thus, detecting quality difference effects, for example, should require far fewer students than access effects.

With regards to the access hypothesis, we made the assumption throughout this paper that students who have access to the textbook would use the textbook in effective ways. Of course, access is not a guarantee for learning. A student with access to a textbook could easily choose to ignore it or engage in ineffective learning strategies. These students are no better off with a textbook than they were without one. This fact creates a general boundary condition on the ability for access alone to affect learning. Practically speaking, the effect of access on learning depends critically on usage after access to the materials is supplied. If students engage with the book in ineffective ways, then access will be an irrelevant factor. To this end, we simply caution readers that while access is an important step towards improving learning, it not sufficient.

### Limitations

It is important to point out that our simulated experiments provide only a proxy measure of statistical power. In particular, these simulations estimate power under an unrealistically optimistic experimental scenario. The situation only gets more difficult in real world studies, which have instructor effects, student effects, and other confounds to control for. These variables only add noise to the data, and reduce this probability of success even further. Thus, a researcher hoping to estimate their statistical power with a real-world data should understand that their actual power will be lower than those shown in Fig 2.

Another limitation of our analysis of past research studies is that we assumed an effect size *d* of 0.25, rather than computing the observed effect sizes post hoc. Unfortunately, as previously mentioned, the vast majority of research we reviewed did not report sufficient statistics to conduct such analysis. If the true effect size of OER adoption is larger, then these studies may have had considerably more statistical power than what we estimated. To this end, we conducted a supplemental analysis which estimates the minimum effect size required in order for an OER study of varying sample sizes and access rates to achieve an acceptable level of power. This analysis is explained in detail in S1 Appendix, and the results shown on S1 Fig. Should additional research become available that suggests the effect size is different than the one we used, S1 Fig can be used to determine whether power of these past studies was adequate. Also, we remind readers that the source code of our analysis is available such that anyone rerun our analysis with varying levels of *d*.

### Relevance to educational research

While it is tempting to think that the research failings discussed in this paper are unique to OER, the reality is that these failings are the result of a common mistake in educational research (and even social science research more broadly). Specifically, that mistake is overgeneralizing the influence of an experimental variable without critically considering the context in which that variable is manipulated. The importance of contextual factors was articulated decades ago by [33, 34], who noted the fragile nature of many of the most landmark findings in memory research (e.g., levels of processing [35]). In particular, it was observed that minor changes to an experimental design could completely change the outcome of a manipulation. The critical insight of [33] was that variables not manipulated by the experimenter are just as important as the ones that were manipulated. The materials used, the final assessment, the types of students, and the interactions among all these factors were all critically important. Thus, if one wants to understand whether an intervention affects learning, they need to be aware of the context in which that intervention is taking place.

Of course, researchers and practitioners are naturally compelled to focus only on variables of interest in isolation. To illustrate, one of the most influential studies in education is a meta-analysis of over 800 factors that affect student learning [36]. While compiling such an extensive list of factors is quite the achievement, in our view, it presents an unrealistic view about the nature of learning. It leads one to a misplaced belief that certain techniques are better than others. However, even the strongest factors listed by [36] could quite easily be rendered ineffective by applying them to certain topics, certain populations of students, or certain outcome measures. For example, it is well known that effectiveness of an educational strategy or intervention can depend on the prior aptitude of individual’s in a study [37]. The very existence of such interactions prevents us as a field from ever discovering “laws” of human learning [38] or making broad sweeping claims about any intervention. In sum, the effectiveness of any educational intervention will almost always depend on the context in which it is implemented.

Failing to consider the importance of context can lead to poor study design and misleading conclusions. In this paper, we discussed the importance of student access in moderating the effectiveness of OER. Past researchers assumed OER would have a general effect on learning and failed to context influences, which lead to a dearth of under powered and ill designed studies. A similar analogue comes from the oft maligned enterprise of *media comparison studies* [39–42]. Media comparison studies typically evaluate student learning from a standard instructional strategy delivered on different types of “media” (e.g., computer vs. paper). Like OER, most of these studies have produced null results, and have been vehemently criticized for decades as being without merit [39]. Indeed, [39] took a strong stance that media is only a vehicle for delivering instructional strategies, and that media itself will never influence learning. While this is often true, others [41, 42] have argued that many media comparison studies employed standardized research designs that were not well suited to measure the unique mechanisms afforded by media evaluated in the study. [43] reviews a wide variety of media studies which reveal the nuances of when media can have a meaningful influence on student outcomes. Thus, by carefully considering the context in which an intervention occurs and is evaluated and devising appropriate hypothesis to test, one can design studies that effectively and appropriately measure the unique merits of the intervention.

## Conclusions

The goal of educational research is to answer important questions about education through scientific analysis. However, studies that are not grounded in theory or lack statistical power do not provide meaningful insights for answering these questions. On the contrary, such studies only muddy the waters, and move us further from determining the truth. Despite the large number of studies that have been conducted on OER efficacy, these studies unfortunately do not provide much information about the potential impacts of OER on student learning. While the large number of null effects may suggest that OER adoption may not provide much benefit to student learning, the reality is these studies do not provide much insight, because they were incapable of detecting positive effects even if they did exist. As it currently stands, the question of whether OER affects student learning remains unanswered.

## Supporting information

S1 Appendix Supplemental simulation analysis. (PDF)

S1 Fig Minimum effect size required to detect an effect of OER at 80% success rate as a function of access rate and sample size. For a given value of *a*, the minimum value of *d* necessary to detect an effect of OER is very sensitive to sample sizes *n* below 1000. Conversely, for a given value of *n*, the minimum value of *d* is extremely sensitive to the access rate.
(TIFF)
