# Supplementary material for: Do open educational resources improve student learning? Implications of the access hypothesis
Source: PLoS One. 2019 Mar 6;14(3):e0212508. doi: 10.1371/journal.pone.0212508 (PMC6402753; doi:10.1371/journal.pone.0212508)
Supplement: S1 Appendix — (PDF) [file pone.0212508.s001.pdf]

S1 Appendix. Supplemental Simulation Analysis

In our primary simulation, we examined the impact of sample size and access rate on the overall statistical power of a typical OER experiment. In doing this, we assumed a fixed effect size d of 0.25 for the impact of having a textbook. Of course, as we noted previously, the true effect size for this parameter is unknown, and very well could be smaller or larger than this value. A slightly different way of approaching the problem is to determine how large the effect size would have to be in order to reliably detect an effect of OER, given a certain sample size and access rate. In this supplemental simulation, we examine the impact of d itself on statistical power.

Methods

Design

Despite the different problem formulation, our supplemental simulation design is identical to the primary simulation study

Parameter Values

Our parameter values are identical to those in the primary simulation study with the exception of the effect size d, which is determined as part of the simulation for each value of n and a.

Procedure

For each level and n and a, we determined the minimum effect size d necessary to detect an effect of OER with a statistical power (the probability of rejecting the null hypothesis) greater than or equal to .8. It is generally accepted that .8 is an acceptable level of statistical power. To do this, we discretized our search space to all potential values of d between 0 and 3 in increments of .01. We then employed a binary search to find the smallest possible d that achieved an 80% success rate or higher. We employed 10,000 simulations at each point in the binary search when estimating statistical power.

Results

The results are shown on S1 Fig. The minimum effect size d required to detect the effect of OER decreases both as n increases or as the access rate a decreases. We find that the minimum value of d is highly sensitive to sample size when n is smaller than 1000. Further, the minimum value of d is very sensitive with respect to the OER access rate a, and this sensitivity increases as the access rate approaches 1. As a concrete example, a study of with a sample size n of 3000 and access rate of a of .4 requires only a modest effect size d of around .25. As the access rate increases to .8, the necessary effect size grows to .5 and at an access rate of .9 the minimum effect size is greater than 1.
