# Supplementary figures and images for: Do open educational resources improve student learning? Implications of the access hypothesis
Source: PLoS One. 2019 Mar 6;14(3):e0212508. doi: 10.1371/journal.pone.0212508 (PMC6402753; doi:10.1371/journal.pone.0212508)

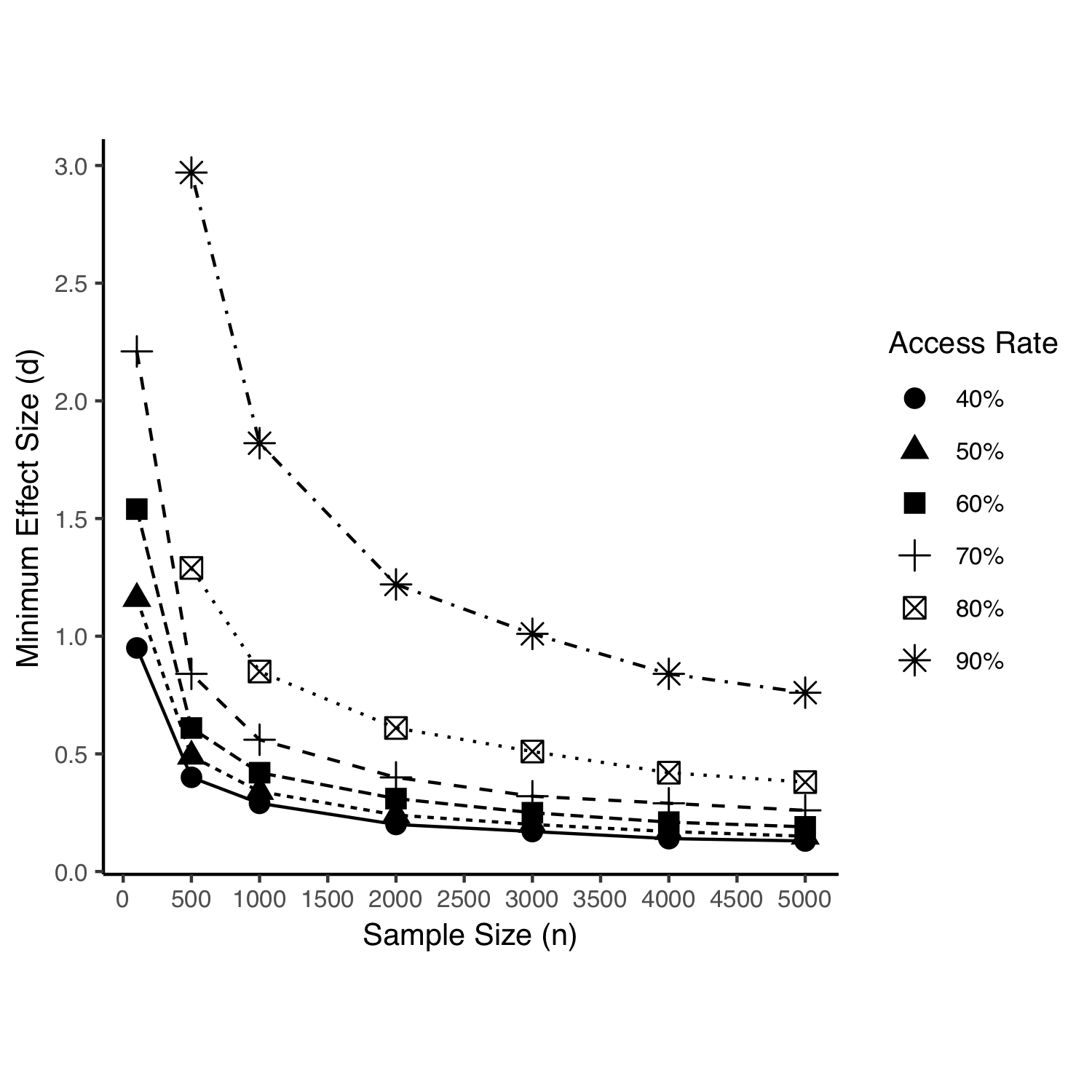

Supplement: S1 Fig — For a given value of a, the minimum value of d necessary to detect an effect of OER is very sensitive to sample sizes n below 1000. Conversely, for a given value of n, the minimum value of d is extremely sensitive to the access rate. (TIFF) [file pone.0212508.s002.tiff]
